# 

**Published:** 1881-12

**Authors:** 


					﻿Whol^/Number,
QCXXXIII.
DECEMBER, 1881.
Number 5,
Volume XXI.
THE
BUFFALO
Medical and Surgical Journal.
EDITORS:
THO8. I.OTHROP, M. D.,
I». W. VAN I’EYMA, M.
A. K. DAVIDSON, M.
IAJCIKIN HOWH, M. !>.,
HERMAN MVNTER, 91. I>.
BUFFALO:
Published by the Medical Journal Association.
Read the Notice of this Journal among the Advertisements.
Entered at the Post-Office at Buffalo, N. Y., as Second-Class Mail Matter.
				

